# A Rare Case of Suicide by Ingestion of Phorate: A Case Report and a Review of the Literature

**DOI:** 10.3390/healthcare9020131

**Published:** 2021-01-29

**Authors:** Angelo Montana, Venerando Rapisarda, Massimiliano Esposito, Francesco Amico, Giuseppe Cocimano, Nunzio Di Nunno, Caterina Ledda, Monica Salerno

**Affiliations:** 1 Legal Medicine, Department of Medical, Surgical and Advanced Technologies, “G.F. Ingrassia”, University of Catania, 95123 Catania, Italy; francesco.amico05@community.unipa.it (F.A.); giuseppe.cocimano@you.unipa.it (G.C.); monica.salerno@unict.it (M.S.); 2 Department of Clinical and Experimental Medicine, University of Catania, 95123 Catania, Italy; vrapisarda@unict.it (V.R.); cledda@unict.it (C.L.); 3 Department of History, Society and Studies on Humanity, University of Salento, 73100 Lecce, Italy; nunzio.dinunno@unisalento.it

**Keywords:** phorate, acute toxicity, chronic toxicity, histopathological kidney, toxicological examination

## Abstract

Phorate is a systemic organophosphorus pesticide (OP) that acts by inhibiting cholinesterases. Recent studies have reported that long-term low/moderate exposure to OP could be correlated with impaired cardiovascular and pulmonary function and other neurological effects. A 70-year-old farmer died after an intention ingestion of a granular powder mixed with water. He was employed on a farm for over 50 years producing fruit and vegetables, and for about 20 years, he had also applied pesticides. In the last 15 years, he used phorate predominantly. The Phorate concentration detected in gastric contents was 3.29 µg/mL. Chronic exposure to phorate is experimentally studied by histopathological changes observed in the kidney. In the light of current literature, our case confirms that there is an association between renal damage and chronic exposure to phorate in a subject exposed for years to the pesticide. Autopsies and toxicological analyses play a key role in the reconstruction of the dynamics, including the cause of the death.

## 1. Introduction

Phorate (IUPAC name: O,O-diethyl S-[(ethylsulfanyl)methyl] phosphorodithioate; CAS Number: 298-02-2) is a systemic organophosphorus pesticide (OP) that acts by inhibiting cholinesterases, which are the enzymes involved in transmitting nerve impulses [1]. Phorate is highly toxic to birds, fish, and mammals (male rat oral LD50 for the metabolite phorate oxon = 0.88 mg/kg) [2], and accidental human exposure, resulting in death in some instances, has been reported [3].

The self-poisoning of organophosphorus pesticide is a major clinical and public health problem across many rural regions. Some studies have highlighted higher suicide rates among farmers than the general population [4,5,6]. Some reviews pointed out higher suicide rates among farmers than any other occupational group in the United Kingdom and in Australia [7,8,9]. In contrast, applicators in Italy had a lower rate of accidents and suicide [10]. In fact, Parrón et al. [11] showed a suicide rate in certain areas of Spain where there was greater use of phorate [12], and ecological and case studies suggested an association between organophosphate pesticide (OP) use and suicide [13].

The Agricultural Health Study (AHS) is a large, prospective cohort study of private pesticide applicators (mostly farmers), designed to study associations between cancer and other chronic diseases and farm-related exposures [14]. Pesticides are associated with the following cancer: lung cancer [15,16], pancreatic cancer [17], colon and rectal cancer [18,19] all lymphohematopoietic cancers [20] leukemia [21], Non-Hodgkin lymphoma [22], multiple myeloma [23], bladder cancer [24] brain cancer and melanoma [25,26].

In humans, chronic exposure to phorate has been shown to cause a reduced acetylcholinesterase (AChE) activity in both blood plasma and the brain [27]. Its mechanism of action consists in the inhibition of AChE activity by phosphorylating the serine hydroxyl group of the substrate-binding domain [28]. In cases where there is an accumulation of acetylcholine (ACh), the “cholinergic syndrome” can develop, which causes an overstimulation of nicotinic, muscarinic, and central ACh receptors; at the level of the central nervous system (CNS), this causes different effects such as headache, drowsiness, dizziness, confusion, blurred vision, slurred speech, ataxia, coma, and convulsions, until there is a block of the respiratory center [29,30,31]. Recent studies have highlighted that long-term low/moderate exposure to OP could be correlated with impaired neurobehavioral function or other neurological effects [32,33], while the studies have reported higher suicide rates among farmers who used it than in the general population [34,35]. Other investigations on the chronic toxicity of OP have shown kidney damage in animal models mediated by several mechanisms such as damage to the cell membrane and proteins through oxidative stress induced by the generation of free oxygen radicals, functional disturbances related to plasma membrane injury, cellular DNA damage, and activation of apoptosis-related p53 [35,36,37,38,39,40,41,42,43]. In the present study, we report a case of suicide carried out through the high ingestion of phorate of an agricultural worker exposed to phorate, at low doses, for several years. However, there is no evidence for an association between nephrotoxicity and chronic long-term exposure to low levels of phorate exposure in humans in the literature. Through this case report and a systematic review of animal and human models, this study aims to correlate kidney damage with chronic exposure to pesticide and phorate in the subjects exposed for years to the pesticide and dead after an intentional ingestion of phorate.

## 2. Materials and Methods

### 2.1. Case Description

A 70-year-old farmer was found disoriented and sweaty by his daughter. The man had been drinking a granular powder mixed with water. She immediately alerted the emergency services, but the man suddenly collapsed during transport to the emergency department. Despite resuscitation maneuvers, he died. He had been a farmer for over 50 years producing fruit and vegetables, and for about 20 years, he had used phorate. In the last nine years, he had used predominantly phorate twice/month, for 6–7 working h/day from March to July. From his medical records, compiled by his family doctor, it appears that the worker had had a history of depression for 10 years that had been treated pharmacologically with a specialist prescription.

No ethical committee was required. Written informed consent was obtained from the relatives.

### 2.2. Autopsy Findings

**Figure 1:**
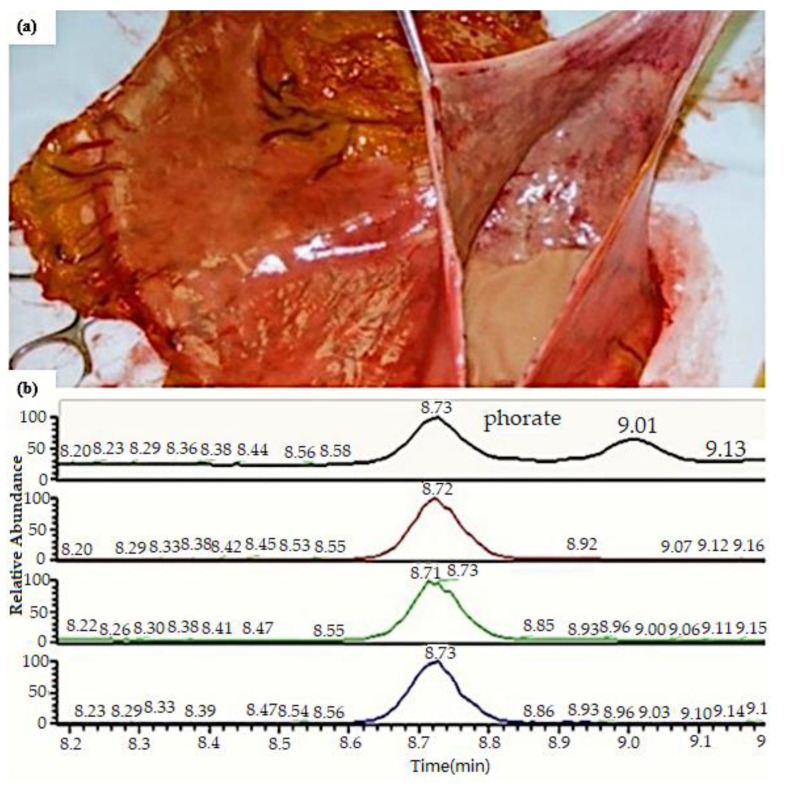
(**a**) the stomach’s opening: stomach contained 100 mL of brown color; (**b**) chromatogram of the case.

A complete autopsy was performed 48 h after death. External examination of the body showed a robust physique (height 165 cm, weight 81 kg), with hypostasis on the upper half of the body (head, neck, superior thorax, and upper limbs). The autopsy revealed the heart with a regular shape, weight 470 g, and measured 12 × 9.5 × 6.8 cm; the coronary arteries were healthy with a right dominance. The myocardium and the valvular apparatus were normal. The analysis of the respiratory system was performed through the removal of the heart-lung block. The larynx was edematous; the lung parenchyma appeared increased in volume, heavy (left 430 g, right lung 500 g), the consistency was emphysematous during palpation with congestion and petechiae. The esophagus walls had a brownish coloration; the stomach contained 100 mL of brown liquid, which was sampled (see Figure 1a). Organs taken during the autopsy showed evident signs of diffused visceral congestion. The organ specimens were fixed in 10% buffered formalin and embedded in paraffin. Microscopy with hematoxylin-eosin staining was performed: the brain showed intraparenchymal haemorrhages, perineuronal and perivasal oedema. The lungs displayed subpleural and endoalveolar haemorrhages, massive endoalveolar edema, and scattered bronchopneumonia outbreaks; there was an infiltrate of peribronchiolar lymphocytes and neutrophils were detected in the lungs and laryngeal mucosa.

Specimens of biological fluids (blood, urine, and gastric contents) taken during the autopsy were frozen (−20 °C). Sections of tissue were set up with hematoxylin and eosin for histological examination.

### 2.3. Poisoning

To determine what the subject had ingested, gastric contents were collected at the time of autopsy and stored at −20 °C, and 1 mL of samples was analyzed, after liquid/liquid extraction, by gas chromatography–mass spectrometry (GC–MS) analysis (single quadrupole).

### 2.4. Chronic Exposure Assessment

In the absence of chronic pre-mortem exposure data, the subject’s kidney sections, after H&E staining, were observed to detect the presence of damage due to chronic exposure to phorate to verify if there had been an extended exposure during life.

### 2.5. Systematic Review

In accordance with the PRISMA statement a systematic review was performed [16].

### 2.6. Literature Search

SCOPUS, Medline (using PubMed as the search engine), Embase, and Web of Sciences databases were searched up to 30 September 2020 for the association of toxic phorate exposure with suicide and histopathological kidney changes in humans as the primary outcome.

MeSH was used with the following entry terms: “Phorate” AND “death”; “Phorate” AND “suicide”; “Phorate” AND “kidney”. A search of the identified manuscripts was then made for inclusion suitability in this systematic review, and the research papers of significance were collected and reviewed.

### 2.7. Inclusion and Exclusion Criteria

The following criteria were used: (1) Studies that evaluated the correlation between Phorate and death (suicide, kidney damage, animals and humans). The following exclusion criteria were then used: (2) animal studies, (3) original articles in non-English language; and (4) editorials, posters, abstracts.

For duplicate studies, the article with detailed information was included.

### 2.8. Quality Assessment and Data Extraction

Two reviewers, A.M. and C.L. processed articles independently. The title, abstract, and full text of each potentially pertinent study was reviewed. Through the consultation and debate of additional reviewer V.R., any divergence on the eligibility of the studies was determined. The following information was extracted and organized from all suitable papers: authors, year of publication, the nationality of subjects, and study characteristics.

### 2.9. Characteristics of Eligible Studies

After a search of the scientific literature by reviewers, a total of 15 documents were collected.

**Figure 2:**
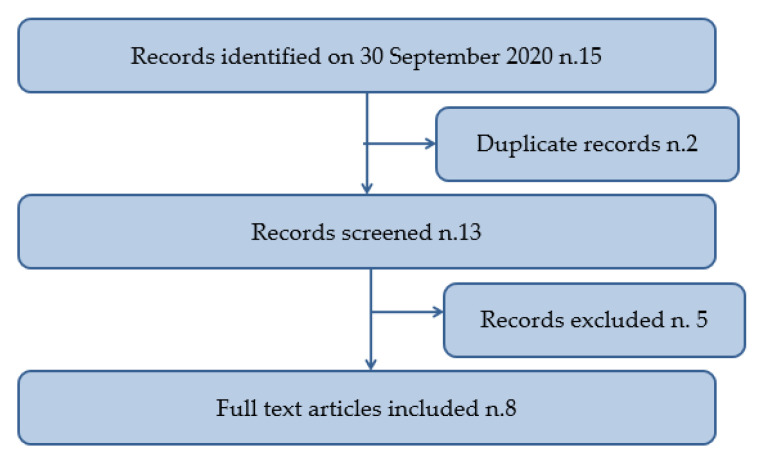
Flow diagram illustrating included and excluded studies in this systematic review.

Two were excluded after a subsequent review of the title and abstract, and 5 studies were ruled out after a review of the manuscript. In conclusion, 8 studies satisfied totally the inclusion criteria and were included in the systematic review. A flowchart depicting the choice of studies is shown in Figure 2.

A summary of the details of the included research papers is reported in Table 1.

### 2.10. Outcomes of Eligible Studies

From the eight studies, only two were carried out on humans; both were case reports of acute exposure.

Peter et al. [44] reported a case of a 30-year-old woman who, after a family dispute, swallowed 50 mL of phorate. She survived after gastric lavage. Khatiwada et al. [3] reported a case of a 30-year-old woman and her 80-year-old grandmother who unintentionally ingested phorate granules mistaken for food (sesame seeds). The 30-year-old woman was pronounced dead on arrival at the emergency department; the 80-year-old woman survived after resuscitation procedures.

The other six studies were conducted on animals and related chronic exposure to phorate. The experiments were conducted on rats that were given only phorate or with other pesticides for a period from a minimum of 14 days to a maximum of 24 weeks consecutively. Due to chronic exposure, these rats showed kidney and DNA damage.

## 3. Results

In our case, phorate concentration detected in the gastric contents was 3.29 µg/mL (see Figure 1b).

According to the macroscopic and microscopic findings, the cause of death was attributed to respiratory failure with pulmonary dysfunction due to an acute cholinergic crisis. The larynx was edematous; the lung parenchyma appeared increased in volume, and the consistency was emphysematous during palpation. The esophagus walls had a brownish coloration; the stomach contained 100 mL of brown liquid, which was sampled. Histological examination of the lungs revealed a generalized stasis; it was observed that inside the lungs, the alveolar spaces were occupied by an eosinophilic proteinaceous material and some hemosiderin-laden macrophages associated with passive congestion. The use of a higher power microscope allowed us to see intra-alveolar edema and alveolar capillaries’ engorgement.

**Figure 3:**
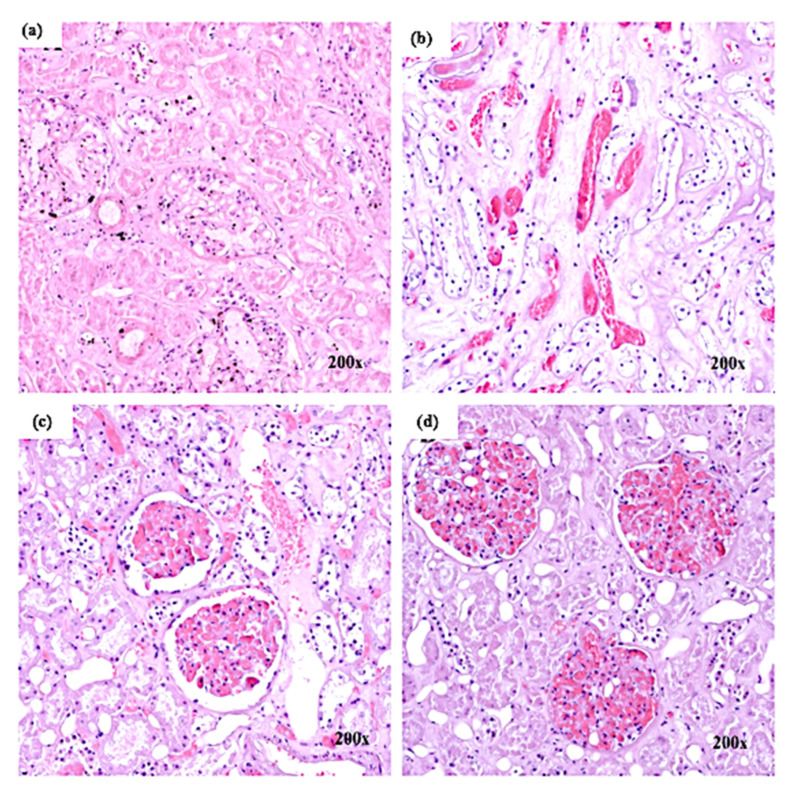
Histological findings in the kidney: (**a**) glomerulus hypercellularity; (**b**) deposition of eosin-positive material in the tubules; (**c**) increased space between Bowman’s capsule and glomerulus; (**d**) increased cellularity in the glomerulus.

The glomerulus exhibited hypercellularity (see Figure 3a). At certain places, leucocytic infiltration was noticed. There was a deposition of eosin-positive material between the tubules and degenerating tubules (see Figure 3b). A shrunken of the glomeruli were seen in some areas. Thus more spaces between Bowman’s capsule and the glomerulus were displayed (see Figure 3c). There was increased cellularity in the glomerulus (see Figure 3d), tightly filling Bowman’s capsule, and dilated tubules were seen with the separation of epithelial cells from the underlying basement membrane. The proximal and distal tubules showed hypertrophy of epithelial cells with obliterated lumina.

## 4. Discussion

In the case reported here, death followed fatal oral ingestion of phorate by a farmer who had used phorate for about 20 years. He had used predominantly phorate twice per month for 6–7 working h/day from March to July in the last nine years.

In the literature, there were never described fatal cases of accidental ingestion of phorate. It was described a case after unintentional ingestion of phorate by a 30-year-old woman in the literature. The woman confused phorate granules for sesame seeds, she mixed it with pickle and rice and consumed it. She was taken to the hospital and, after resuscitation, was transferred to the intensive care unit. She was extubated on the 17th day and discharged on the 23rd day [3].

In another case report a 28-year-old woman, in India, after quarrelling with her family, impulsively swallowed 50 mL of phorate. Immediately she lost consciousness and was taken to hospital arriving about nine hours after her suicide attempt. After five days of deep coma, the patient started to recover and was fully conscious by day 15 [44].

The reason behind the elevated risk of mood disorder in farming populations is unclear. The neurotoxic effects of high-level acute poisoning are well known and involve inhibition of the enzyme AChE, provoking changes in peripheral, autonomic, and CNS function resulting in a group of physical, cognitive, and psychiatric symptoms. Nevertheless, organophosphorus pesticide (OP) disrupts many other neurotransmitters, and some of these are entailed in mood regulation, such as serotonin. This could clarify the connection between pesticide exposure and mood disorders seen in earlier studies [39,40].

Compared to other occupational groups was reported that workers exposed to OP had a high incidence of depression and anxiety [32,33], but, to the best of our knowledge, our case is the first in which phorate was ingested intentionally.

Deaths from acute OP intoxication usually result from the depression of the CNS respiratory system and respiratory failure caused by a combination of bronchoconstriction and excessive respiratory secretions [41,42].

Chronic renal failure is one of the problems manifested in clinical follow-up of the patients and causes an increase in OP intoxication mortality [43]. In this specific case, after the exposition for several years to phorate, the patient developed depressive disorders, and the postmortem histological findings showed chronic kidney damage.

A significant number of studies demonstrated that phorate induces structural DNA modification with cellular damage in cultured human and animal cells [50,51]. The production of reactive oxygen species (ROS) in the vicinity of the protein molecule and the strong binding of phorate to the proteins induce the protein damage [52]. Several studies confirmed the ROS generating capability of phorate both extracellularly and intracellularly, with mitochondrial damage. In fact, the production of ROS induced mitochondrial damage, which suggests phorate toxicity in exposed cells [53,54]. Damage to lysosomal membranes is known to release lysosome protease into intracellular spaces, affecting neighboring cells, and triggers cell death due to necrosis [55].

These structural cell alterations have been highlighted in the kidney, a target organ of phorate as highlighted both in animal and human studies. In general acute renal failure is due to the oxidative stress, where OP directly damages renal tubules and renal parenchyma, and to the myoglobinuria caused by muscle fasciculation [56,57,58].

Chronic exposure to phorate is experimentally studied by histopathological changes observed in the proximal renal tubules; similar changes were observed in experiments conducted on animal and human models [33,34].

Qi et al. [46] investigated the protective effect of quercetin against the combined toxic action induced by the mixture of four organophosphate pesticides (dimethoate, acephate, dichlorvos, and phorate) given to the rats for 90 days, using metabonomics detected in rat urine. The authors showed the toxicity induced by chronic exposure to low-level mixture of OPs on the metabolism of fatty acids, energy and sex hormones, antioxidant defense system, DNA damage, and kidney function.

In a very similar study, Du et al. [46] in the plasma rats carried out a metabolomic analysis of the combined toxic action of long-term low-level exposure to a mixture of four organophosphate pesticides (dichlorvos, dimethoate, acephate, and Phorate) given for 24 weeks. The results highlighted that the mixture of OP pesticides induced oxidative stress, renal dysfunction, disturbed the metabolism of lipids and amino acids, and the thyroid gland. Observational histopathological changes in the kidney are marked by renal tubular epithelial cell swelling and granular degeneration, which were observed in the low-dose group 12 weeks after treatment.

Histopathological changes in the kidney were seen in the high- and middle-dose groups than those in the low-dose group 12 weeks after treatment. The main characteristic findings were the renal tubular epithelial cell swelling, granular degeneration, and vacuolar degeneration.

The same study was carried out by Sun et al. [47], analyzing the urine of rats investigating the toxic effect of long-term and low-level exposure to phorate using a metabolomics approach ultra-performance liquid chromatography-mass spectrometry (UPLC-MS). Male Wistar rats were administered low doses of 0.05, 0.15, or 0.45 mg/kg body weight (BW) phorate daily through water for 24 weeks consecutively. The levels of creatinine (CR) and urea nitrogen (BUN) were significantly elevated in the high-dose group, indicating kidney damage after exposure to phorate.

Li et al. [48] examined the effect of quercetin against a mixture of four organophosphates (dichlorvos, acephate, dimethoate, and phorate) inducing nephrotoxicity in rats, given for 90 days. Similar to the previous studies, the histopathological examination performed on the kidney sections (H&E method) showed extensive cell vacuolar denaturation and desquamation of the epithelial lining of the tubules, renal damage by impairing the reabsorption capacity of the proximal tubules, and by decreasing glomerular filtration rate.

Mohssen [49], in his study, evaluated biochemical (serum creatinine) and histopathological kidney alterations in male Swiss albino mice, Mus musculus, caused by subchronic inhalation of the recommended field dose of Phorate (20 kg/ha). In particular, from the second to twelfth week of exposure, the kidney, in the cortical tubules, showed at the beginning widespread cloudy degeneration, with a few foci of hydropic degeneration until the blockage of the lumen by necrotic cells. In the interstitial spaces, red blood cells and macrophages were present in the first weeks, with an increase of the chronic inflammatory infiltrate associated with foci of hemorrhage in the cortex. Kidney damage is caused by the ability of phorate to induce inflammation-inducing the formation of reactive oxygen, damage to the mitochondria, damage to the cell membrane and proteins, including enzymes, which finally result in loss of membrane fluidity and function. These alterations are associated with an early injury to cellular membranes after exposure to different toxins that induce plasma membrane injury disturbances.

Moreover, phorate exhibits cellular DNA damage and activation of apoptosis-related p53, caspase 3 and 9 genes, inducing the generation of intracellular ROS that induce reduced activities of the antioxidant enzymes catalase (CAT), glutathione (GSH) and lipid peroxidation (LPO), which were observed in the kidney of rats exposed to phorate [36]. Male Wistar rats exposed to phorate at varying oral doses of 0.046, 0.092, or 0.184 mg phorate/kg BW for 14 days showed, after histological investigations of tissues, changes in kidney function such as renal blood flow, concentrating substances, and biotransformation of the parent compounds make this tissue sensitive to a variety of toxins. The greater Bowman’s space showed with infiltration of renal parenchyma by inflammatory leukocytes, dilated blood vessels, and renal necrosis; similar results were shown by Mohssen [49].

## 5. Conclusions

Through this case and the data reported in the literature, the authors want to point out that: (a) the use of OP and phorate should be limited in time because increases symptoms of psychological distress, including suicidal thoughts; (b) the OP, particularly after the prolonged exposition, have a toxic effect on the kidney that is histologically notable; (c) in this case, the cause of death was attributed to respiratory failure with pulmonary dysfunction due to an acute cholinergic crisis; (d) phorate induces DNA structural alterations and cellular damage in cultured human cells and in animal cells determining qualitative changes in tissues in the kidney.

Moreover, since there are, to date, few reports of similar deaths, our report provides useful information regarding this particular kind of death. All aspects of the forensic death investigation triad -investigation (history), pathology, and laboratory results—are essential and must be evaluated in context with one another. In this regard, the toxicological investigation was decisive, allowing us to identify the stomach’s substance and was analyzed after liquid/liquid extraction by GC–MS analysis (single quadrupole).

Since extremely hazardous neurotoxic pesticides are still ordinally used in developing countries, further research on mental health is crucial to make an important preventive action by governmental and international bodies.

## Figures and Tables

**Table 1:** Characteristics of eligible studies (animals and humans).

Reference	Study Design	Target	Exposure	Intervention/Outcome	Main Findings
Khatiwada et al., 2012 [3]	case report	n.2 women: (1) 30-year-old (2) 80-year-old	acute	unintentional ingestion of phorate granules mistaken for food (sesame seeds)	30-year-old woman dead on arrival at the emergency department; 80-year-old woman survived after resuscitation procedures.
Peter et al., 2008 [44]	case report	30-year-old female	acute	impulsively swallowed 50 mL of phorate after a family dispute	survived after hospital treatment.
Qi et al., 2017 [45]	prospective, observational	rats; urine analysis	Chronic	rats were given a mixture of four op pesticides (dimethoate, acephate, dichlorvos, and phorate) for 90 days.	Alteration of kidney function, modification of DNAwith alteration of the metabolism of fatty acids, energy and sex hormones, antioxidant defense system.
Du et al., 2014 [46]	prospective, observational	rats; the plasma was analyzed	chronic	mixture of four op pesticides (dimethoate, acephate, dichlorvos, and phorate) for 24 weeks	kidney damage of tubular cell, granular and vascular degeneration.
Sun et al., 2012 [47]	prospective, observational	rats; metabonomics evaluation of urine by uplc-ms; long-term and low-level exposure	chronic	phorate daily in drinking water at low doses of 0.05, 0.15 or 0.45 mg/kg body weight (bw) for 24 weeks consecutively	kidney damage: the levels of creatinine (cr) and urea nitrogen (bun) were significantly elevated in the high-dose group, indicating kidney damage after exposure to phorate.
Li et al., 2016 [48]	prospective, observational	rats; the authors examined the effect of quercetina	chronic	mixture of four organophosphates (dichlorvos, acephate, dimethoate and phorate) for 90 days	kidney damage: histopathological examination showed extensive cell vacuolar denaturation and desquamation of the epithelial lining of the tubules; renal damage by impairing the reabsorption capacity of the proximal tubules and by decreasing glomerular filtration rate.
Mohssen 2001 [49]	prospective, observational	rats	chronic	subchronic inhalation of the recommended field dose of phorate (20 kg/ha).	kidney damage: impairment of glomerular function and tubular damage with mild to severe multifocal cloudy and hydropic degeneration (edema) with necrosis in kidney tubules.
Saquib et al., 2012 [50]	prospective, observational	rats	chronic	14 days of varying oral doses of phorate of 0.046, 0.092 or 0.184 mg	kidney damage: infiltration of leukocytes in the bowman’s space with dilated blood vessels, and renal necrosis.

## Data Availability

All data are included in the main text.
